# Supplementary material for: The Prevalence and Correlates of Disability in Singapore: Results from a Nationwide Cross-Sectional Survey
Source: Int J Environ Res Public Health. 2021 Dec 11;18(24):13090. doi: 10.3390/ijerph182413090 (PMC8701250; doi:10.3390/ijerph182413090)
Supplement: Supplementary file 1 [file ijerph-18-13090-s001.zip › ijerph-1481060-supplementary.pdf]

Table S1: Prevalence of specific disability (standard threshold) by socio-demographic factors

	Vision (%)	Hearing (%)	Mobility (%)	Cognition (%)	Self-care (%)
**Age groups (years)**	Disability	Disability	Disability	Disability	Disability
18 to 34	0.3	0.0	0.1	1.0	0.0
35 to 49	0.0	0.4	0.7	0.5	0.0
50 to 64	1.4	0.0	2.8	0.2	0.5
65 and above	1.9	1.1	5.6	0.2	0.2
**Gender**					
Female	0.6	0.0	1.9	0.5	0.1
Male	0.9	0.5	1.7	0.5	0.3
**Ethnicity**					
Chinese	0.8	0.3	1.5	0.4	0.1
Malay	0.6	0.1	3.4	1.0	0.4
Indian	0.6	0.1	2.2	0.6	0.2
Others	0.0	0.0	0.7	1.4	0.5
**Education**					
Primary and below	1.6	0.8	6.3	0.3	0.7
Secondary	2.0	0.0	1.7	0.1	0.1
Pre-U/Junior College	0.0	0.0	1.8	0.6	0.3
Vocational Institute/ITE	0.0	0.0	0.5	1.9	0.1
Diploma	0.0	0.6	0.0	1.2	0.0
Degree, professional certification, and above	0.0	0.0	0.1	0.2	0.0
**Marital Status**					
Single	0.4	0.0	0.5	1.2	0.1
Married/Cohabiting	0.6	0.4	1.8	0.3	0.0
Separated/Widowed/Divorced	3.3	0.1	6.0	0.2	1.5
**Children**					
Yes	1.0	0.4	2.6	0.3	0.3
No	0.3	0.0	0.4	0.9	0.1

**Employment status**					
Employed	0.3	0.4	0.4	0.6	0.0
Economically Inactive	1.8	0.0	4.6	0.4	0.7
Unemployment	2.7	0.0	8.3	0.4	0.3
**Monthly income SGD (Personal)**					
Below 2,000 (Reference)	1.5	0.2	3.4	0.9	0.4
2,000 to 5,999	0.3	0.2	0.5	0.4	0.0
6,000 and above	0.0	0.8	0.1	0.2	0.0
No income	1.4	0.1	3.7	0.2	0.3
**Monthly Income SGD (Household)**					
Below 10,000	1.0	0.3	2.4	0.7	0.2
10,000 to 19,999	0.0	0.0	0.1	0.2	0.1
20,000 to 39,999	0.0	0.0	0.0	0.0	0.0
40,000 to 79,999	0.0	6.7	0.0	0.0	0.0
80,000 to 99,999	0.0	0.0	0.0	4.0	0.0
100,000 and above	0.0	0.0	0.0	0.0	0.0
No income	6.9	0.0	0.4	0.0	0.0
**Lifestyle**					
Healthy Lifestyle	0.8	0.5	1.9	0.4	0.4
Unhealthy Lifestyle	0.7	0.1	1.7	0.6	0.06
**Chronic conditions**					
No chronic condition	0.4	0.6	0.3	0.3	0.0
One chronic condition	0.7	0.0	0.5	1.0	0.1
At least two or more chronic conditions	1.0	0.0	5.5	0.5	0.6
**Physical Activity**					
Sufficiently active MET >= 600	0.7	0.3	0.8	0.5	0.0
Insufficiently active MET < 6000	0.9	0.0	6.6	0.4	0.9
**Mean PCS (S.E)**	48.5 (3.5)	46.7 (1.3)	36.1 (2.2)	46.5 (1.6)	24.1 (6.9)
**Mean MCS (S.E)**	46.7 (1.8)	50.2 (3.0)	50.2 (2.3)	44.6 (2.7)	56.3 (7.1)

ITE: Institute of Technical Education; MET: Metabolic Equivalents; MCS: Mental Component Summary Score; OR: Odds Ratio; PCS: Physical Component Summary Score

Table S2: Prevalence of overall disability (wider threshold) by socio-demographic groups, lifestyle and chronic conditions

	Overall Disability	
	No disability	Disability
	(n) %	(n) %
**Age groups (years)**		
18 to 34	633 (80.1)	190 (20.0)
35 to 49	540 (73.9)	179 (26.1)
50 to 64	478 (64.8)	296 (35.2)
65 and above	242 (45.6)	337 (54.4)
**Sex**		
Female	905 (67.2)	569 (32.8)
Male	988 (71.0)	433 (29.0)
**Ethnicity**		
Chinese	547 (69.5)	249 (30.5)
Malay	583 (63.5)	391 (36.6)
Indian	617 (72.1)	301 (27.9)
Others	146 (72.9)	61 (27.1)
**Education**		
Primary and below	287 (52.7)	350 (47.3)
Secondary	422 (61.8)	262 (38.2)
Pre-U/Junior College	96 (78.6)	30 (21.4)
Vocational Institute/ITE	189 (69.7)	78 (30.3)
Diploma	344 (72.5)	135 (27.5)
Degree, professional certification, and above	555 (81.4)	147 (18.6)
**Marital Status**		
Single	528 (76.9)	203 (23.1)
Married/Cohabiting	1225 (68.0)	635 (32.1)
Separated/Widowed/Divorced	139 (50.9)	164 (49.1)

**Employment status**		
Employed	1399 (74.0)	534 (26.0)
Economically Inactive	419 (56.6)	410 (43.4)
Unemployment	75 (59.8)	58 (40.2)
**Monthly income in SGD (Personal)**		
Below 2,000	663 (58.1)	573 (41.9)
2,000 to 5,999	749 (75.8)	267 (24.2)
6,000 and above	245 (79.6)	55 (20.4)
No income	133 (62.1)	86 (37.9)
**Lifestyle**		
Healthy Lifestyle	949 (72.9)	421 (27.2)
Unhealthy Lifestyle	944 (66.3)	579 (33.7)
**Chronic conditions**		
No chronic condition	955 (76.9)	274 (23.1)
One chronic condition	508 (72.1)	258 (27.9)
At least two or more chronic conditions	429 (53.8)	463 (46.2)
**Physical Activity**		
Sufficiently active MET >= 600	1655 (71.2)	774 (28.8)
Insufficiently active MET < 6000	236 (58.1)	228 (41.9)

n represents the sample observations
ITE: Institute of Technical Education; MET: Metabolic Equivalents; SGD: Singapore Dollars
Missing values: Marital Status (n for “No disability” = 1), Monthly Personal Income (n for “No disability” = 103, n for “Disability” = 21), Lifestyle (n for “Disability” = 2), Chronic conditions (n for “No disability” = 1, n for “Disability = 7), Physical Activity (n for “No disability” = 2)
